# Metagenomic analysis of gut bacteria in different developmental instars of *Spodoptera litura*

**DOI:** 10.1128/spectrum.02081-25

**Published:** 2026-03-06

**Authors:** Jiayun Yao, Changli Yang, Hancheng Wang, Changyu Zhang, Jianyu Meng

**Affiliations:** 1 Guizhou Provincial Key Laboratory for Tobacco Quality Improvement and Efficiency Enhancement, College of Tobacco, Guizhou University 71206, Guiyang, Guizhou, China; 2 Guizhou Provincial Key Laboratory for Agricultural Pest Management of the Mountainous Region, Institute of Entomology, Guizhou University 71206, Guiyang, Guizhou, China; 3 Guizhou Tobacco Science Research Institute 499147 , Guiyang, Guizhou, China; National Center for Genetic Engineering and Biotechnology, Khlong Luang, Pathum Thani, Thailand

**Keywords:** *Spodoptera litura*, metagenomics, developmental instars, metabolism

## Abstract

*Spodoptera litura* is a globally distributed agricultural pest across Asia and Australia (EPPO database, https://gd.eppo.int/taxon/PRODLI/distribution), whose gut microbiota significantly influences host feeding, digestion, immunity, and development. We used whole metagenomic sequencing to analyze the diversity and functional roles of gut bacteria at different developmental stages (eggs, first to sixth instar larvae, pupae, and adults). Findings revealed that Pseudomonadota predominated at the phylum level, with notable differences across instars: Bacillota was dominant in young larvae, whereas Verrucomicrobiota was added in older larvae, eggs, pupae, and adults. At the genus level, *Pseudomonas* dominated, *Enterococcus* was prevalent in larvae, and *Piscirickettsia* was prevalent in eggs, pupae, and adults. Metagenomic analysis identified numerous carbohydrate-active enzymes (CAZy database) aiding in plant cell wall polysaccharide digestion. Kyoto Encyclopedia of Genes and Genomes pathway analysis indicated differential gene expression in metabolism and xenobiotic degradation across instars, with metabolic gene annotation levels declining as development progressed. Detoxification-related enzyme genes were predominantly expressed in early instar larvae and adults, uncovering microbial origins of these enzymes.

**IMPORTANCE:**

Our study provides evidence that the gut microbiota significantly modulates the physiology of *Spodoptera litura*, with profound effects on its dietary habits, metabolic processes, and host fitness. Using whole metagenomic sequencing, we analyzed gut bacteria across different life stages. At the phylum level, Pseudomonadota and Bacillota were dominant, while at the genus level, *Pseudomonas* was the most abundant taxon. Metagenomic analysis identified enzymes aiding in plant cell wall digestion. Kyoto Encyclopedia of Genes and Genomes analysis showed varying gene expression in metabolism and detoxification, with higher expression in early instar larvae and adults. This research enhances understanding of *S. litura* gut microbiota-host interactions and supports novel pest control strategies targeting gut microbiota.

## INTRODUCTION

*Spodoptera litura* (Lepidoptera: Noctuidae), a globally significant agricultural pest, is characterized by its polyphagous nature and broad distribution. It infests plants across 109 families, including cotton, legumes, tobacco, and vegetables, often leading to intermittent outbreaks (1–3). This pest exhibits high reproductive potential, strong migratory capacity, and adaptability to diverse ecological conditions (2, 4, 5). Females deposit large egg (E) masses, with young larvae foraging gregariously and older larvae dispersing to consume substantial crop volumes, resulting in significant agricultural losses (6–8). Current management strategies mainly rely on chemical insecticides. However, long-term use has exacerbated insecticide resistance in *S. litura* and contributed to severe food and environmental contamination (9–12).

The insect gut system, which plays a critical role in feeding, digestion, and excretion, operates in a dynamic and variable environment (13, 14). This creates a unique colonization niche for symbiotic microorganisms, most of which aggregate in the digestive tract as gut microbiota (15–19). These microorganisms possess diverse enzyme systems that facilitate vitamin synthesis, fat and carbohydrate absorption, detoxification, and enhanced insecticide resistance (15, 20–22). For instance, in the Lepidopteran pest *Helicoverpa armigera*, gut bacteria such as *Enterococcus* and *Enterobacter* have been demonstrated to mediate the degradation of host plant toxins, thereby facilitating insect adaptation (23). Similarly, the gut microbiota of *Spodoptera frugiperda* can inactivate the insecticide chlorpyrifos via symbiotic enzymes, directly contributing to host pesticide resistance (24). Additionally, *Burkholderia* in the gut of *Riptortus pedestris* assists in degrading fenitrothion and enhances host resistance to this insecticide (25).

Under normal conditions, the symbiotic microbial community within insects remains relatively balanced and stable, exhibiting interdependence and mutual regulation during host growth and development (26–29). Disruption of this balance severely threatens insect health and development. Consequently, gut microbiota have emerged as a promising foundation for developing novel pest control strategies (19, 30–32). By manipulating gut microbiota composition and destabilizing symbiotic microbial balance, environmentally friendly biological control methods can be achieved (33, 34). For instance, Pietri et al. (35) demonstrated that combining antibiotics with insecticides significantly enhances lethality against insecticide-resistant Blattella germanica and reduces their resistance. Another innovative approach involves engineering gut microbiota to express specific dsRNA targeting key pest genes. Whitten et al. (36) genetically modified *Rhodococcus rhodnii* in the gut of *Rhodnius prolixu*s to produce dsRNA targeting the vitellogenin gene, reducing E hatchability and effectively controlling pest populations.

Current research is increasingly focused on controlling *S. litura* through its pathogenic microorganisms (37, 38). The gut microbial community of *S. litura* has been identified as a critical factor in host nutrition metabolism, immune regulation, and environmental adaptation (13, 19, 37). However, the composition and abundance of symbiotic bacteria across different developmental stages of *S. litura* remain unexplored (38, 39). Recent advancements in metagenomics technology have provided a robust platform for analyzing complex microbial communities, offering insights into the composition, diversity, and functional gene expression of insect gut bacteria (40, 41). This approach holds significant potential for elucidating the role of gut microbiota in *S. litura* and developing targeted pest management strategies.

This study aims to elucidate the composition, diversity, and functional gene expression of gut bacteria in *S. litura* across different developmental stages using metagenomic analysis. To achieve this, individuals of *S. litura* at various developmental stages were collected, and their gut contents were extracted for metagenomic sequencing. The resulting data were analyzed using bioinformatics tools to compare the community structure and functional gene profiles of gut bacteria at each stage. This approach reveals the influence of developmental changes on the gut microbial community and explores the potential roles of gut microbes in the growth and development of *S. litura*. By enhancing our understanding of the gut microbial community in *S. litura*, this study provides a scientific foundation for its ecological control and offers innovative strategies and methodologies for biological pest management.

## MATERIALS AND METHODS

### Insect source

Larvae of *S. litura* were originally collected from tobacco fields in Fuquan (26°42'N, 107°31'E), Guizhou province of China, and subsequently reared in a climate-controlled chamber at a temperature of 26°C ± 1°C, a relative humidity of 70% ± 5%, and a light:dark photoperiod of 14 h:10 h. Larvae were reared on an artificial diet described by Zhang et al. (42). Adults (A) were provided with 10% honey solution as a nutritional supplement.

### Extraction of gut contents

The gut samples of first to sixth instar larvae (L1–L6), pupae (P), A, and the entire E were collected for metagenomic analysis. For the E stage, entire E were directly used as samples. Larvae were subjected to a starvation period prior to dissection to minimize contamination from the food bolus and frass. Specifically, L1–L3 and L4–L6 were starved for 4 and 8 h, respectively. Prior to gut dissection, the insects were dipped in 75% ethanol for 30 s for surface sterilization, followed by two sequential rinses with sterile phosphate-buffered saline (PBS) to thoroughly remove residual ethanol. Then, the entire guts were dissected out in PBS droplets on glass slides. The dissected whole guts were then transferred to 1.5 mL centrifuge tubes. Each treatment group was performed in triplicate. Samples were flash frozen in liquid nitrogen and stored at −80°C until further analysis. Sequencing was performed by Novogene Bioinformatics Technology Co., Ltd. (Beijing, China).

### Preprocessing of sequencing data

A metagenomic library of *S. litura* gut microbiota was constructed following the standard Illumina DNA library preparation protocol. A 300-bp insert size paired-end library was prepared and sequenced on the Illumina HiSeq 2000 platform. Raw reads were subjected to quality control processing, including: (i) removal of reads with >40% bases having quality scores ≤5; (ii) exclusion of reads containing >10% unidentified bases (*N*); (iii) elimination of adapter sequences (minimum 15-bp overlap threshold); and (iv) filtration of host-derived sequences through alignment against the *S. litura* reference genome (43–45).

### Metagenomic assembly

Clean data were assembled using MEGAHIT (v1.2.9) with parameters: -k-min 21 -k-max 121 -k-step 20 --min-contig-len 500, and scaffolds from single-sample assembly were filtered to remove fragments <500 bp (43, 46). Statistical analysis and gene prediction were performed, and scaffolds were fragmented at *N* connections to generate “scaftigs.” The length distribution of scaftigs was analyzed and plotted for each sample.

### Species annotation

Open reading frame (ORF) prediction was conducted on scaftigs (≥500 bp) using MetaGeneMark (v3.38) with default parameters for metagenomic sequences, with predictions <100 nt excluded. Redundancy was removed using CD-HIT software (v4.8.1) with a sequence identity threshold of 95% and alignment coverage of 90% to generate a non-redundant initial gene catalog (47). Non-redundant continuous gene-encoding nucleotide sequences were defined as genes.

Clean data from each sample were mapped to the initial gene catalog using Bowtie2 to quantify reads aligned to each gene. Genes with ≤2 reads in any sample were excluded, yielding the final gene catalog (unigene) for downstream analysis (45). Gene abundance was calculated based on aligned read counts and gene length.

Unigenes were aligned against Bacteria, Fungi, Archaea, and Viruses sequences from the NCBI NR (v2023-09) database using DIAMOND (v2.1.6) with blastp option --evalue 1e-5 --max-target-seqs 1 (43, 48). To resolve multiple alignment results at different taxonomic levels, the Lowest Common Ancestor (LCA) algorithm (implemented in MEGAN v6.21.10) was applied. The taxonomic level preceding the first branching point was assigned as the species annotation for each sequence (49).

Relative abundance tables were generated at multiple taxonomic levels (domain, phylum, class, order, family, genus, and species) using LCA annotation results and scaftig abundance. Species annotations were visualized with Krona (50). The top 10 most abundant phyla in each sample were identified, with remaining species grouped as “Others,” and a bar chart of phylum-level annotations was created. Unigenes were aligned to functional databases (Kyoto Encyclopedia of Genes and Genomes [KEGG] version 2023 and CAZy database version 202308) using DIAMOND, and the highest-scoring alignment (one high-scoring segment pair [HSP] > 60 bits) for each sequence was selected for downstream analysis.

### RT-qPCR analysis of detoxification enzyme genes

Expression of two detoxification genes across developmental stages was quantified in gut samples using *EFα1* as the reference gene. Primers were designed with Primer Premier 6 (Table 1) and synthesized by Sangon Biotech (Shanghai). Total RNA was extracted from *S. litura* midgut using Eastep Super Total RNA Extraction Kit, quantified with NanoPhotometer P-Class, and reverse-transcribed with StarScript II First-strand cDNA Synthesis Kit (Genestar). Quantitative real-time PCR (RT-qPCR) was performed using TB Green Premix DimerEraser (TaKaRa) in a 20 μL reaction containing 10 μL premix, 1 μL each primer (0.01 mol/L), 1 μL cDNA, 0.4 μL ROX Reference Dye II, and 6.6 μL diethylpyrocarbonate water. Cycling conditions were 95°C for 30 s, followed by 40 cycles of 95°C for 5 s and 55°C for 30 s.

**TABLE 1:** Primers used for real-time quantitative PCR validation

Gene name	Primer sequences	Annealing temperature	Protein name
*Efα1*	GACTTGGGTAAGAAGAAG GATGACATGGAATGGATG	45	Reference gene
*GST1*	ACACACTGTACCACTGCTGG ATAGCACCCGAGTTTCCTGC	62	Glutathione S-transferases
*GSTe14*	ACGATAGCCACGCGATACTG CGTGATGTGATCTGCAGCGA	62	Glutathione S-transferases
*UGT2B4*	ATGACAACGTACTCGCCCTG ATTGCCGGATACCAAGGACA	62	UDP-glucuronosyltransferase
*UGT2B19*	AAGCCAGTGCCCAGTTTGTA CCGTCCTTCGCGTCATCTAA	62	UDP-glucuronosyltransferase

### Statistical analysis

Experimental data were processed using Excel 2010 and GraphPad Prism 8. The 2^−ΔCt^ method was used for relative quantification of the expression levels of five genes. The mean and standard deviation of ΔCt values were calculated for experimental and control groups. Significance testing was performed using Tukey’s test in SPSS 22.0 software (*P* < 0.05).


ΔCt=meanCtvalueoftargetgene−meanCtvalueofreferencegene

$$\;\;\;\;Relative\;expression=2^{-\Delta Ct}$$

## RESULTS

### Quality and assessment of gut data

Raw sequencing data were obtained from the samples of *S. litura* at different developmental stages: E, L1–L6, P, and A, with data volumes of 20.14 Gb, 20.74 Gb, 19.10 Gb, 19.76 Gb, 20.01 Gb, 19.41 Gb, 19.68 Gb, 19.63 Gb, and 19.07 Gb, respectively. To ensure the reliability of the subsequent analyses, the raw data were pre-processed, and the filtered high-quality clean data (Clean Base) were 20.01 Gb, 20.34 Gb, 18.83 Gb, 19.57 Gb, 19.84 Gb, 19.25 Gb, 19.49 Gb, 19.39 Gb, and 18.86 Gb, and the proportion of the raw data was over 97%, respectively. The percentages of Q20 bases were all above 96.64%, and the percentages of Q30 bases were above 90.72%. The GC contents ranged from 36.60% to 41.93%, indicating the accuracy of the sequencing data (Table S1).

Valid data from the filtered samples of the E, L1–L6, P, and A were assembled using MEGAHIT to obtain 1,206,567,641, 1,160,161,500, 1,163,441,714, 1,234,010,181, 1,227,514,442, 1,227,515,682, 1,213,137,689, 1,134,967,199, and 1,134,067,651 bp Scaftigs, respectively. The total number of scaffolds obtained was 228,786, 514,525, 539,234, 240,173, 253,109, 251,492, 302,754, 473,796, and 497,862, respectively (Table S2). A total of 266,054 ORFs were predicted from the assembled contigs (Table S3). The non-redundant gene catalog (Unigenes) had an average length of 331.91 bp and an average GC content of 45.27% (Fig. S1).

### Metagenomic sequencing analysis of gut microbiota

Metagenomic analysis revealed that gut bacteria in the E, L1–L6, P, and A samples accounted for 17%, 23%, 24%, 25%, 29%, 31%, 43%, 19%, and 19% of the total gut microbiota, respectively. Archaea, viruses, and eukaryotes were present in the lower proportions, all less than 3%. A total of 75 phyla, 125 classes, 242 orders, 462 families, 832 genera, and 1,700 species of microorganisms were identified in the gut of *S. litura*.

**Fig 1:**
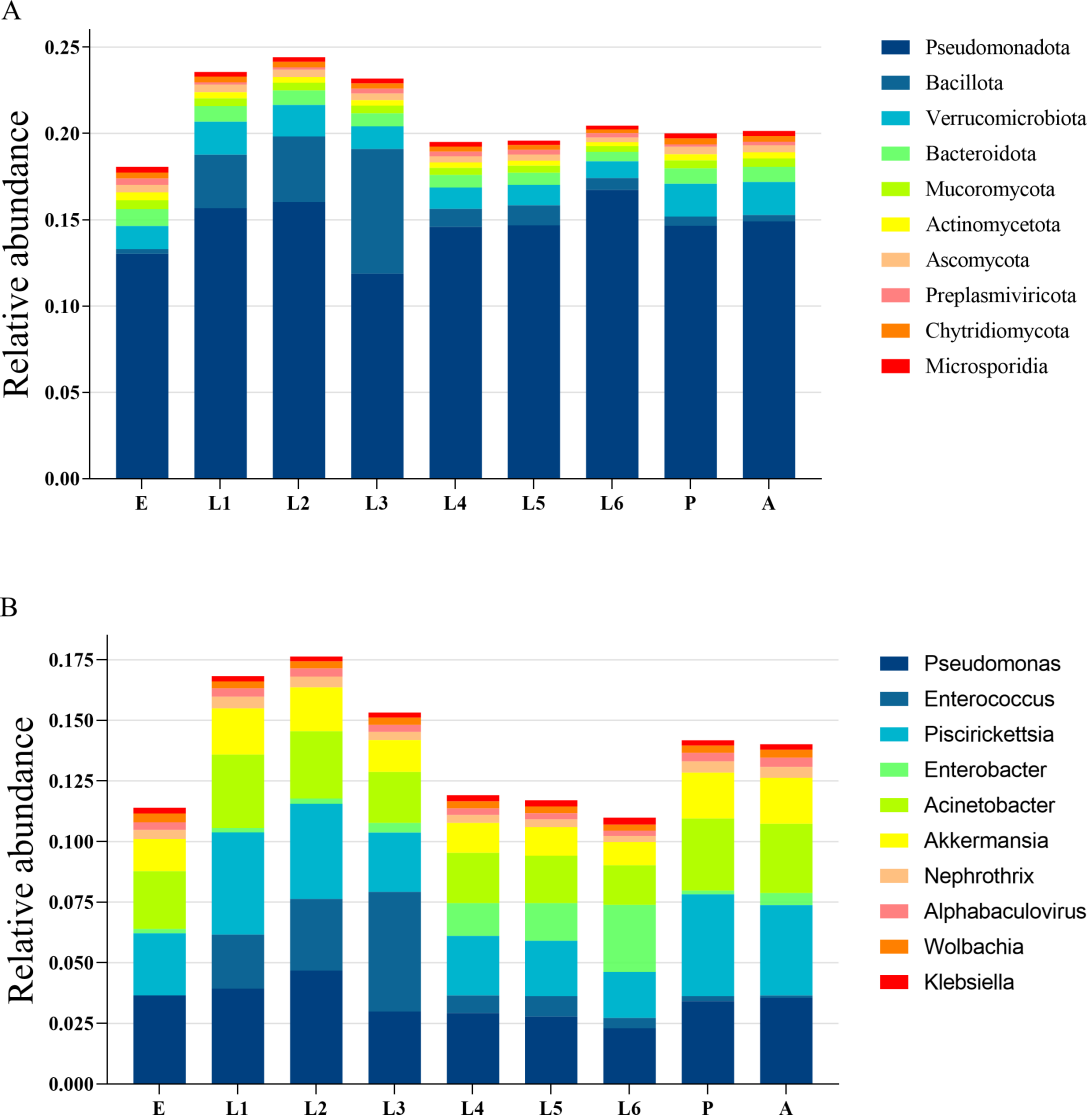
Relative abundance of the top 10 phyla (**A**) and genera (**B**) of intestinal microbial community of *S. litura*.

The top 10 most abundant phyla in each group were selected to create a bar chart of relative abundance (Fig. 1A). In all developmental stages of *S. litura*, the most abundant phylum was Pseudomonadota, followed by Verrucomicrobiota. In the first, second, and third instar larvae stages, the second most abundant phylum was Bacillota. The rest of the annotated top 10 phyla were Bacteroidota, Mucoromycota, Actinomycetota, Ascomycota, Preplasmiviricota, Chytridiomycota, and Microsporidia.

The top 10 most abundant genera in each group were selected to create a bar chart of relative abundance (Fig. 1B). In the developmental stages of *S. litura*, the top 3 most abundant genera in the E, first instar larvae, P, and A stages were *Pseudomonas*, *Piscirickettsia*, and *Acinetobacter*. In the second, third, fourth, and fifth instar larvae stages, the top 3 genera were *Pseudomonas*, *Enterococcus*, and *Acinetobacter*. In the sixth instar larvae stage, the top 3 genera were *Pseudomonas*, *Piscirickettsia*, and *Enterobacter*. The remaining top 10 abundant genera included *Akkermansia*, *Nephrothrix*, *Wolbachia*, *Alphabaculovirus*, and *Klebsiella*.

### Functional annotation of the gut microbiota metagenome of *S. litura*

**Fig 2:**
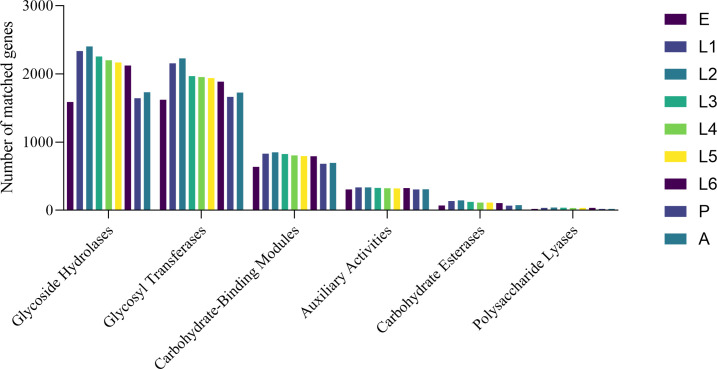
Number of major functional genes in the gut of *S. litura* annotated to the CAZy database.

The CAZy (Carbohydrate-Active enZYmes) database is a specialized database for the study of carbohydrate-active enzymes (CAZymes), covering six major functional classes: glycoside hydrolases (GHs), glycosyl transferases (GTs), polysaccharide lyases (PLs), carbohydrate esterases (CEs), auxiliary activities (AAs), and carbohydrate-binding modules (CBMs). Using the DIAMOND software, unigenes were aligned against the CAZy functional database (Fig. 2). In the gut of *S. litura* at different developmental stages, the most abundant CAZyme genes identified were GHs and GTs, followed by CBMs, AAs, CEs, and PLs. Among the developmental stages, the E, P, and A stages had relatively fewer annotated CAZyme genes, while the first and second instar larval stages had a higher number of annotated genes.

**Fig 3:**
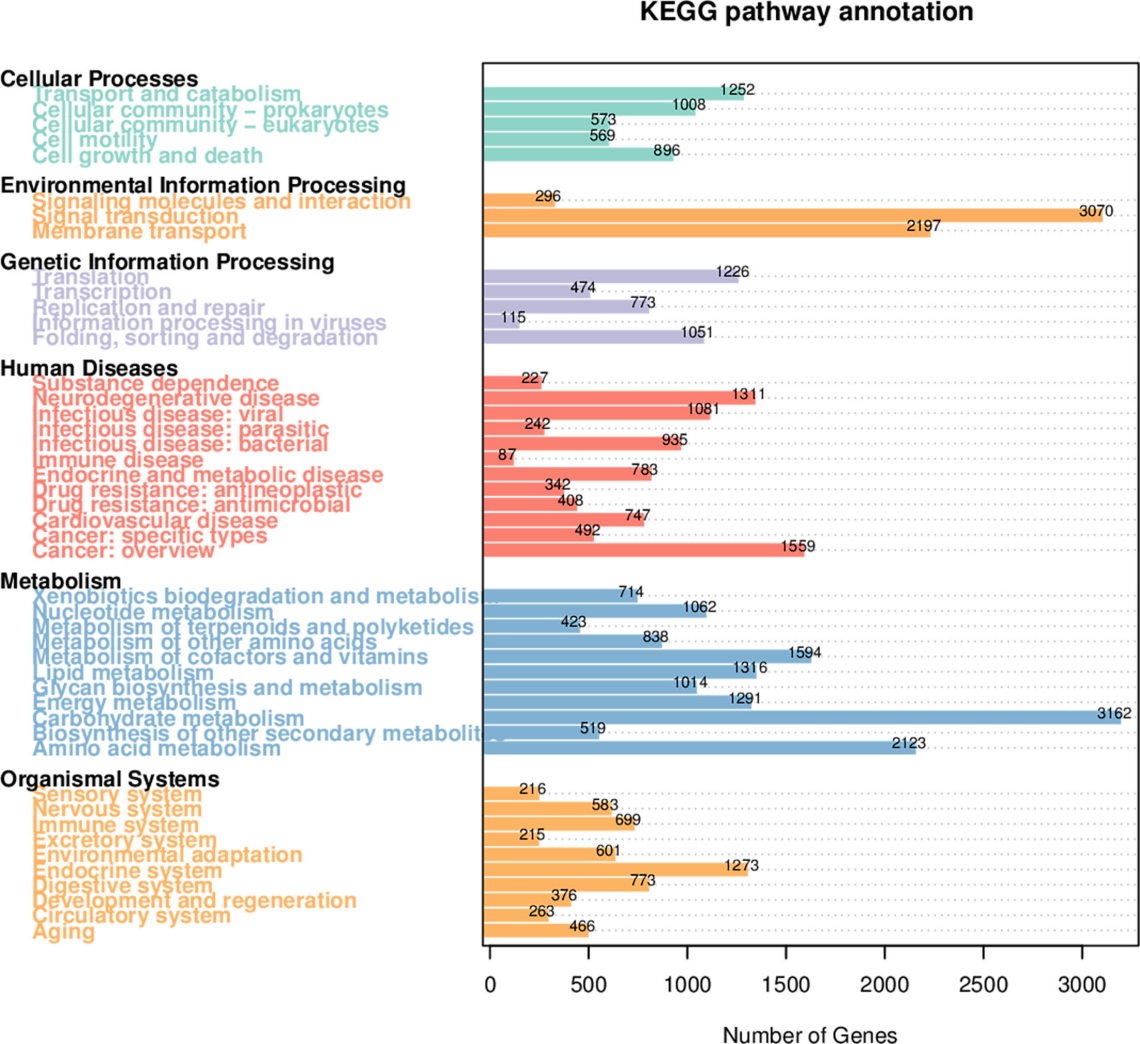
Gene count statistics for KEGG database annotation of *S. litura* gut microbiota.

**Fig 4:**
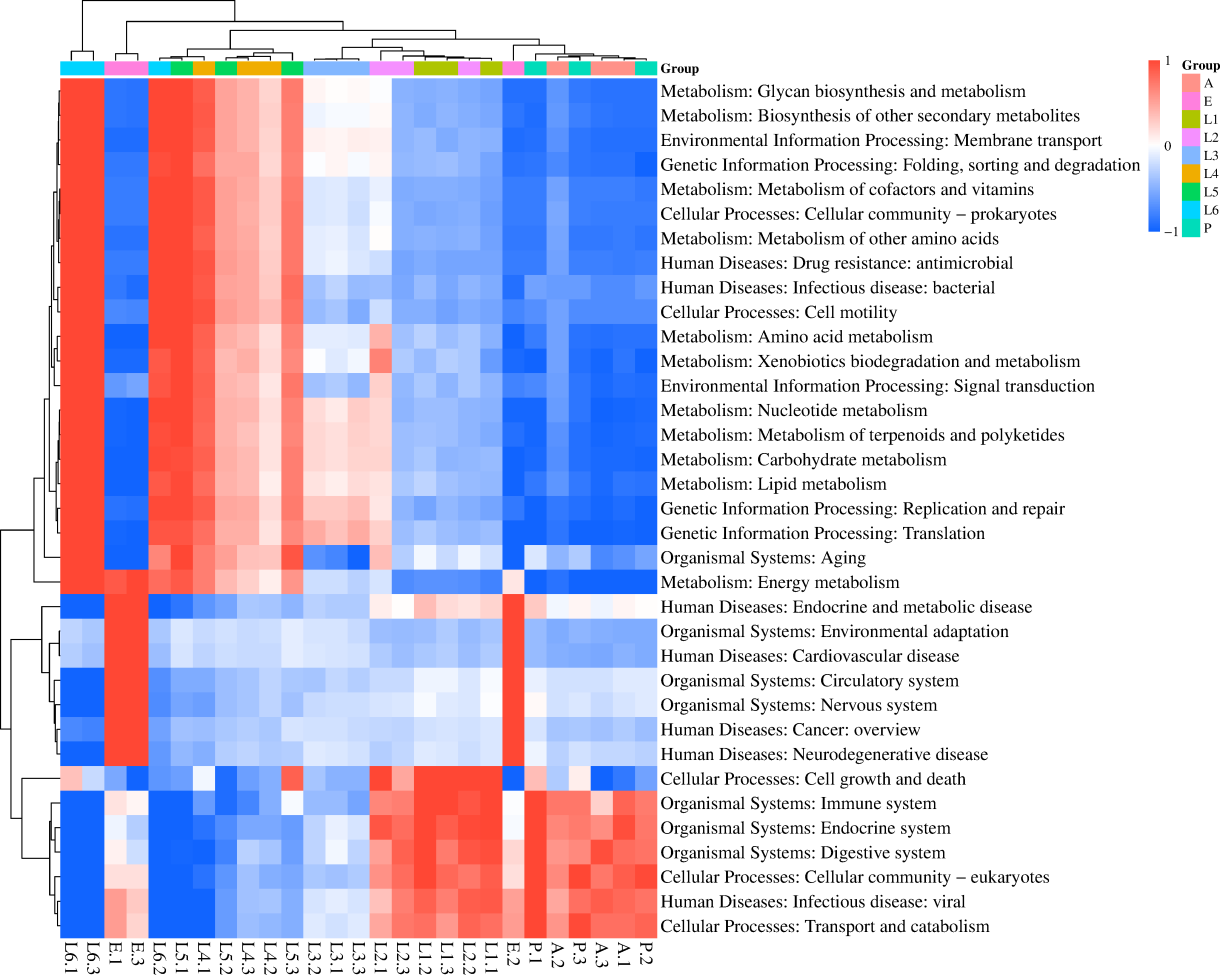
Heatmap of KEGG functional abundance clustering of *S. litura* gut microbiota. The heatmap’s horizontal axis represents sample information, while the vertical axis displays KEGG functional annotations. A functional clustering tree is shown on the left, and the color intensity in the heatmap corresponds to *Z*-scores of normalized relative abundance for each functional category. These *Z*-scores are calculated as the difference between a sample’s relative abundance for a specific function and the mean relative abundance across all samples, divided by the standard deviation of that function’s relative abundance across all samples.

The KEGG is a comprehensive database resource that integrates genomic, chemical, and systemic functional information. Comparing the unigene with KEGG functional database, the number of genes annotated to the six major biometabolic pathways was 3,743 for Cellular processes, 5,372 for Environmental information processing, 3,519 for Genetic information processing, 4,517 for Human diseases, 9,735 for Metabolism, and 3,360 for Organismal systems (Fig. 3). Based on the functional annotations and abundance information from the KEGG database, a heatmap was generated to display the top 35 most abundant functions and their abundance in each sample, with clustering performed to highlight functional differences (Fig. 4). The clustering analysis revealed distinct patterns of KEGG pathway abundance in the gut metagenomes of *S. litura* at different developmental stages. In the E stage, the primary annotated pathways were Cardiovascular disease, Environmental adaptation, Cancer: overview, Neurodegenerative disease, Circulatory system, and Nervous system. In the first and second instar larvae, P, and A stages, the main annotated pathways were Cell growth and death, Transport and catabolism, Infectious disease: viral, Immune system, Endocrine system, and Digestive system. In the third instar larvae stage, the primary annotated pathways were Replication and repair, Translation, Metabolism of cofactors and vitamins, Carbohydrate metabolism, Nucleotide metabolism, and Lipid metabolism. In the fourth, fifth, and sixth instar larvae stages, the main annotated pathways were Signal transduction, Cell motility, Aging, Metabolism of cofactors and vitamins, and Drug resistance: antimicrobial.

**Fig 5:**
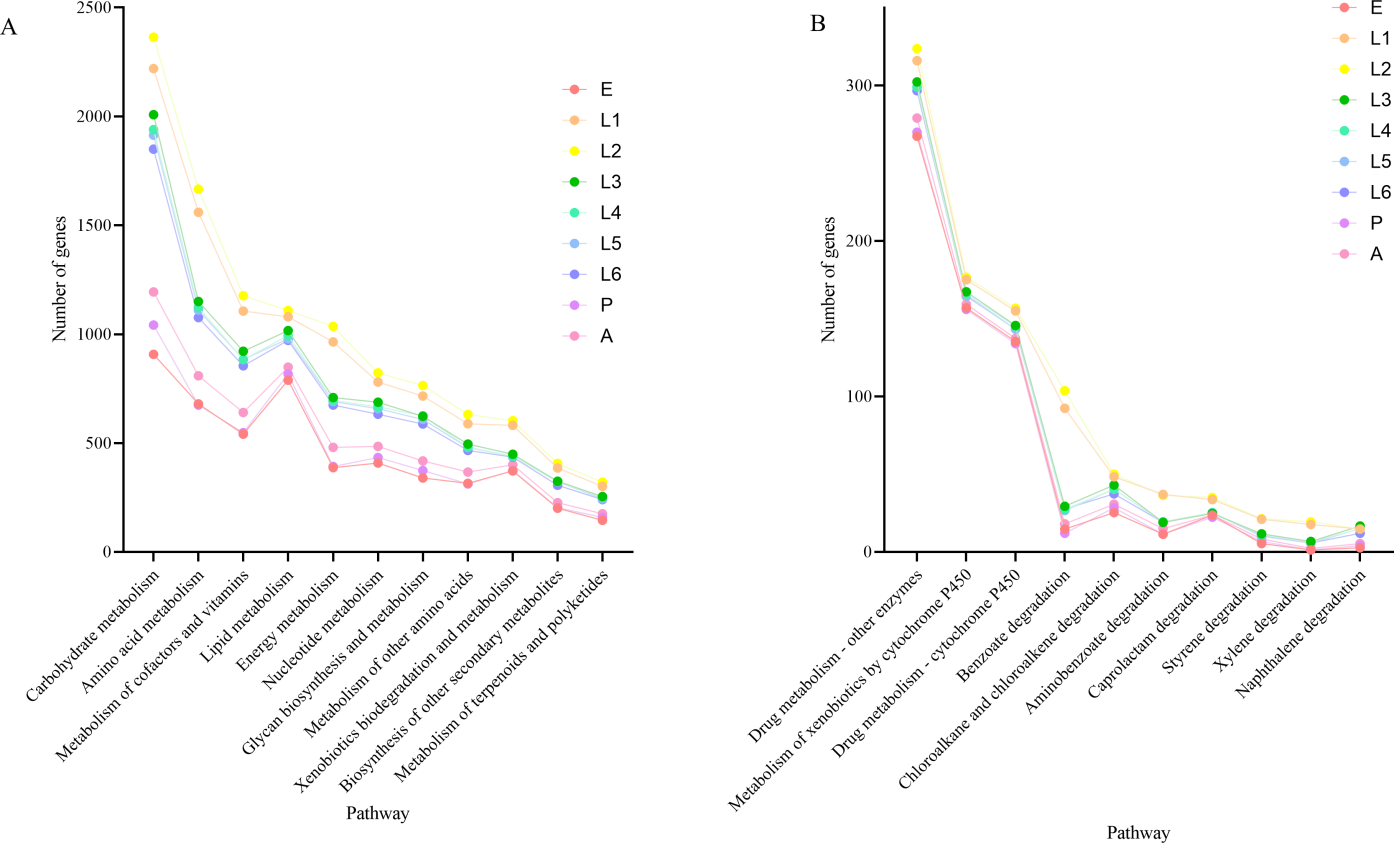
Gene annotation count in 11 sub-pathways of metabolism (**A**) and 19 sub-pathways of Xenobiotics biodegradation and metabolism (**B**) in the gut of *S. litura*.

In the metabolic pathways, the number of annotated genes across different developmental stages, from highest to lowest, was as follows: second, first, third, fourth, fifth, and sixth instar larvae, A, P, and E (Fig. 5A). During the early larval stages, *S. litura* requires substantial metabolic activity to support growth and development. As the insect progresses through its developmental stages, metabolic activity decreases. In the A, P, and E stages, where feeding is minimal or non-existent, metabolism is at its lowest.

Given the polyphagous nature of *S. litura* and its well-documented exposure to diverse plant secondary metabolites and environmental pesticides, we specifically focused on the xenobiotic biodegradation and metabolism functional subgroup to investigate the potential role of the gut microbiota in host detoxification and ecological adaptation. The biodegradation and metabolism of xenobiotics are one of the 11 sub-pathways within the metabolism pathway (Fig. 5B). In the gut bacteria of *S. litura*, 19 sub-functions under the biodegradation and metabolism of xenobiotics pathway were annotated, with the most annotated pathways being Drug metabolism—other enzymes, Metabolism of xenobiotics by cytochrome P450, and Drug metabolism—cytochrome P450. Further analysis of these sub-pathways identified various enzyme genes involved in these metabolic processes. In the Drug metabolism—other enzymes pathway, 22 enzyme genes were annotated, mainly including XDH (xanthine dehydrogenase), NDK (nucleoside diphosphate kinase), and UCK (uridine-cytidine kinase). These enzymes play important roles in the host’s metabolic processes, especially in detoxification and energy metabolism. In addition, the enzyme identified in the Metabolism of xenobiotics by cytochrome P450 pathway was CBR1 (carbonyl reductase 1), whereas the enzyme identified in the Drug metabolism—cytochrome P450 pathway was aofH. The common enzymes across these pathways include ADH5 and frmA (alcohol dehydrogenase 5). The enzymes shared by all three pathways are UGT (UDP-glucuronosyltransferase) and GST (glutathione S-transferase). The metagenomic analysis allowed us to identify the putative microbial sources of the key detoxification enzymes (Table S4). XDH is mainly encoded by *Gammaproteobacteria*, while NDK and UCK are encoded by *Enterococcus*. ADH5 and frmA are encoded by *Enterococcus*. Additionally, adhP is primarily encoded by *Enterobacter* and *Enterococcus*, while GST is jointly encoded by *Pseudomonas*, *Enterobacter*, and *Escherichia*, and UGT is encoded by *Escherichia*. Moreover, these enzyme genes exhibit significant expression differences across the developmental stages of *S. litura. UGTs*, *GSTs*, *XDHs*, *NDKs*, and *CBR1s* are mainly expressed in the A stage, while adhPs are predominantly expressed in the first, second, and third instar larvae stages. *UCKs* and *ADH5s* are primarily expressed in the first and second instar larvae stages.

### RT-qPCR analysis of detoxification enzyme genes

**Fig 6:**
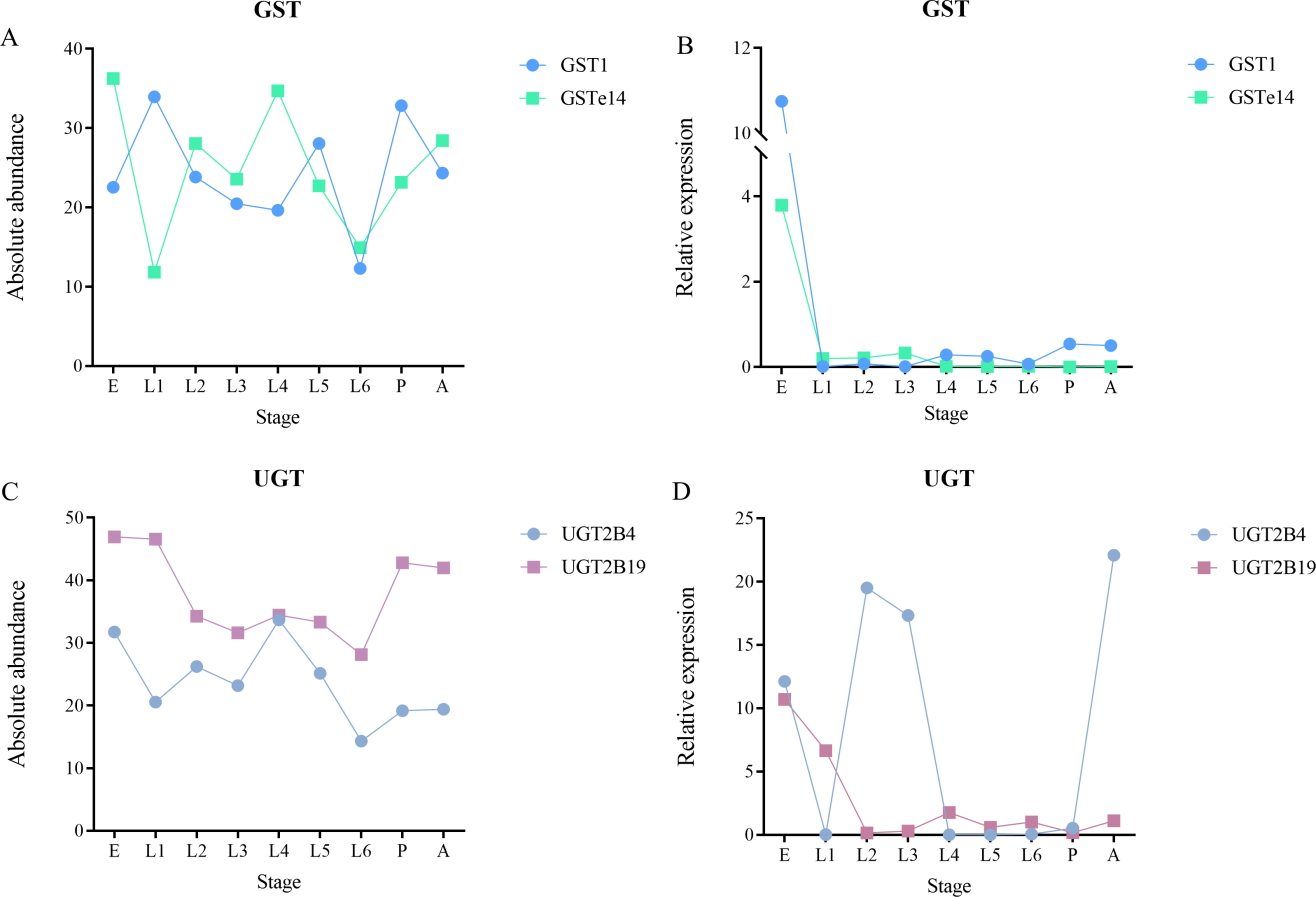
Absolute abundance and relative expression levels of detoxification enzyme genes in the gut across developmental stages. (**A**) GST gene absolute abundance. (**B**) GST gene relative expression. (**C**) UGT gene absolute abundance. (**D**) UGT gene relative expression. Relative expression was quantified using the 2^−ΔCt^ method.

Four detoxification enzyme genes were selected for RT-qPCR analysis (Fig. 6). The results showed that different genes within the same enzyme family exhibited similar expression trends in the gut across developmental stages. When compared with metagenomic absolute abundance patterns, GST genes displayed inconsistent trends, whereas UGT genes were largely consistent with the metagenomic data.

## DISCUSSION

In this study, we revealed the dynamic characteristics of the gut microbial community in *S. litura* at different developmental stages through metagenomic analysis of gut bacteria. A total of 75 phyla, 125 classes, 242 orders, 462 families, 832 genera, and 1,700 species of microorganisms were identified in the gut of *S. litura* at various developmental stages. We analyzed the diversity of microorganisms at different phyla and genera levels across these stages and conducted functional annotation of gut microbiota using the CAZy and KEGG databases. Our findings indicated that the composition and function of the gut microbiota varied significantly among different developmental stages, closely related to the growth and development phases of the insect. This dynamic variation likely reflects the adaptive adjustments of gut microbiota to different host developmental stages. The gut microbiota suggests an adaptation to host development: elevated *Enterococcus* and GHs in early larvae support rapid growth, while increased glutathione S-transferases in later stages may represent a shift toward detoxification during dispersal and feeding.

The gut microbiota of insects is also influenced by the host’s developmental stages (51). A study by Anneleen et al. (52) showed that the dominant bacterial communities in bumblebee larvae were Enterobacteriaceae and Lactobacillaceae, while the core microbiota in A bees consisted of five different types. Our study also found differences in the dominant gut bacterial communities across different developmental stages of *S. litura*: an analysis of gut bacterial phylum diversity at each developmental stage revealed that Pseudomonadota was widely distributed across all stages, with Bacillota dominating in the early instar larval stages, and Verrucomicrobiota significantly increasing in abundance in the late instar larvae, E, P, and A stages. In terms of bacterial genus annotation results, the gut bacterial community of *S. litura* was mainly composed of *Pseudomonas*, with *Enterococcus* being predominant in larvae and *Piscirickettsia* in E, P, and A stages. Devi et al. (53) isolated *Enterococcus casseliflavus*, *Enterococcus mundtii*, *Serratia marcescens*, *Klebsiella pneumoniae*, *Paralactobacillus*, and *Pantoea brenneri* from A *S. litura*. Xia (54) isolated bacteria from the gut of fourth instar larvae of *S. litura* in taro leaf populations and diet populations, and *S. litura* reared on taro leaves had gut biota mainly comprised of *Enterobacteriales* and *Lactobacillales*, while those reared on artificial diet predominantly contained *Pseudomonadales* and *Enterobacteriales*. Similarly, our study also found that *Pseudomonas*, *Enterococcus*, and *Enterobacter* were the dominant genera in the gut of *S. litura* and were widely present in the gut microbial communities at each developmental stage. However, there were also some differences that some bacteria previously reported as dominant gut microbiota in *S. litura* were not detected in this study, which may be due to the differences in the living environment, food types, developmental stages, and research methods of *S. litura*.

Using the CAZy database, we identified a series of CAZymes and found that GHs and *GTs* genes were highly expressed in the gut of *S. litura* at all developmental stages, followed by *CBMs*, *AAs*, *CEs*, and *PLs* genes. Among them, the first and second instar larvae stages had more annotated CAZyme genes, while the E, P, and A had fewer annotated genes. CAZymes are mainly responsible for the digestion of carbohydrates in the insect gut, breaking down complex carbohydrates into simple sugars for easy absorption and utilization by insects (17, 19, 55–57). Termites utilize their gut symbionts and related CAZymes to digest lignocellulosic materials to obtain energy and nutrients (58–62). Certain bacteria in the gut of millipedes can encode GH genes, which are involved in the degradation of plant cell walls, helping millipedes obtain nutrients from plant residues (63). As a herbivorous insect, *S. litura* also contains a large number of CAZymes in its gut, which are abundant during the larvae stages. Since *S. litura* mainly feeds during the larvae stages, CAZymes in its gut play a role in breaking down carbohydrates and digesting food, with GH and GT being the key enzymes.

During their survival, insects face various xenobiotics, including plant secondary metabolites, insecticides, and other environmental toxins (64–67). To adapt to these chemical stresses, insects have evolved complex biodegradation and metabolic pathways: xenobiotic biodegradation and metabolic pathways (68–73). Our research found that in the 19 sub-pathways under the xenobiotic biodegradation and metabolic pathways of the *S. litura* gut, the most annotated pathways were Drug metabolism—other enzymes, Metabolism of xenobiotics by cytochrome P450, and Drug metabolism—cytochrome P450, all of which are related to drug resistance. In addition, we also identified different enzymes involved in these pathways. The Drug metabolism—other enzymes pathway mainly includes XDH, NDK, and UCK; the Metabolism of xenobiotics by cytochrome P450 pathway includes CBR1; and the Drug metabolism—cytochrome P450 pathway includes aofH. The latter two pathways share enzymes such as frmA and adhP, while all three pathways share enzymes such as UGT and GST. These enzymes play important roles in host metabolism, especially in detoxification and energy metabolism (57, 67, 74–76). RT-qPCR analysis revealed that different members within the same detoxification enzyme family exhibited highly similar expression patterns across developmental stages, indicating coordinated transcriptional regulation. However, significant discrepancies were observed between these expression trends and metagenomic absolute abundance: GST gene transcriptional activity was decoupled from gene copy number, possibly due to post-transcriptional regulation or functional redundancy, whereas UGT expression was strongly correlated with abundance, suggesting that its function is more directly controlled by gene dosage. These results demonstrate that metagenomic data alone cannot accurately evaluate the detoxification capacity of the insect gut, and transcriptomic data must be integrated to comprehensively elucidate its functional dynamics.

Further analysis also found that *Enterococcus*, *Enterobacter*, *Escherichia*, *Gammaproteobacteria*, *Vibrio*, *Paenibacillus*, and *Pseudomonas* were involved in the encoding of these enzyme systems, which were not among the most abundant taxa in the overall community (Fig. 1). Studies have found that these bacteria have detoxification metabolic functions (77–79). For example, *E. casseliflavus* can regulate amino acid metabolism and activate the detoxification enzyme activity of the host *Clanis bilineata*, thereby enhancing the insect’s resistance to insecticides (80). *Enterobacter cloacae* strain ECsp1 can significantly upregulate proteins related to xenobiotic degradation pathways, thereby improving the host’s metabolism of secondary metabolites and pesticides (81). *Pseudomonas aeruginosa* can detoxify trinitrotoluene and glyphosate through bio-oxidation, converting them into less toxic compounds (82–84). This functional redundancy, where both dominant and rare microbes contribute to essential pathways, may enhance the metabolic flexibility and adaptive capacity of the host. However, there are few reports on the detoxification function of gut bacteria in insects at present.

For these gut bacteria in *S. litura*, we need to further explore how they participate in the metabolism of toxic substances to reveal their specific mechanisms and functions. In addition, the abovementioned enzyme genes showed significant expression differences in the developmental stages of *S. litura*, mainly expressed in the first, second, and third instar larvae and A stages. The first, second, and third instar larvae and A of *S. litura* are in the key stages of growth and development of *S. litura*. On the one hand, the high expression of detoxification and metabolic enzymes helps insects reduce the negative impact of toxic substances on insect growth and development (76, 85–87); on the other hand, insects in these developmental stages may face more environmental pressures, such as plant secondary metabolites in food and external chemical substances (57, 73). The high expression of detoxification and metabolic enzymes helps insects adapt to these pressures and enhance their survival ability (88–90). In summary, in addition to playing an important role in food digestion and nutrient supply, the gut bacteria of *S. litura* also participate in the detoxification and metabolic processes of *S. litura*, helping *S. litura* degrade plant secondary metabolites, environmental pollutants, and pesticides, thereby enhancing the survival ability of the host.

These findings provide potential targets for drug resistance management, and the development of drug resistance may be delayed by monitoring the expression of key enzyme genes or interfering with the function of specific symbiotic bacteria. In addition, the high expression of detoxicating enzyme in 1–3 instar larvae and A suggests that these stages are sensitive windows for chemical control, and low-dose pesticides and enzyme inhibitors can be combined to improve the control efficiency. Symbiotic bacteria with detoxification function can also be transformed into engineering bacteria to degrade pesticide residues or repair polluted environment, but their large-scale application needs to solve the problems of strain stability and ecological risk assessment.

### Conclusion

This study reveals the composition characteristics and potential functions of the gut microbial community of *S. litura* at different developmental stages, showing that the gut microbial community of *S. litura* has high metabolic diversity and functional complementarity. *Pseudomonas*, *Enterococcus*, and *Enterobacter*, as dominant genera, may play important roles in the host’s nutritional and detoxification metabolism. Future research will further explore the specific functional mechanisms of these bacterial strains in the growth and development of *S. litura*, providing a theoretical basis for developing pest management strategies based on microorganisms.

## Data Availability

This metagenomic project has been deposited in GenBank under accession no. PRJNA1257937.

## SUPPLEMENTAL MATERIAL

Tables S1 to S4 and Figure S1 spectrum.02081-25-s0001.pdf Table S1: Metagenome data of intestinal bacteria of *S. litura*. Table S2: Metagenome assembly results of intestinal bacteria of *S. litura*. Table S3: Statistics of basic information of gene catalog. Table S4: Microbial sources of key detoxification and metabolic enzymes. Figure S1: Unigene length distribution statistics.

ASM does not own the copyrights to Supplemental Material that may be linked to, or accessed through, an article. The authors have granted ASM a non-exclusive, world-wide license to publish the Supplemental Material files. Please contact the corresponding author directly for reuse.
