# Supplementary material for: Metagenomic analysis of gut bacteria in different developmental instars of Spodoptera litura
Source: Microbiol Spectr. 2026 Mar 6;14(4):e02081-25. doi: 10.1128/spectrum.02081-25 (PMC13055308; doi:10.1128/spectrum.02081-25)
Supplement: Tables S1 to S4 and Figure S1 — Table S1: Metagenome data of intestinal bacteria of S. litura. Table S2: Metagenome assembly results of intestinal bacteria of S. litura. Table S3: Statistics of basic information of gene catalog. Table S4: Microbial sources of key detoxification and metabolic enzymes. Figure S1: Unigene length distribution statistics. [file spectrum.02081-25-s0001.pdf]

Supplementary Tables

TABLE S1 Metagenome data of intestinal bacteria of *S. litura*

Sample ID	Raw Base(G)	Clean Base(G)	Clean Q20(%)	Clean Q30(%)	Clean GC(%)	Effective (%)
E.1	7.41	7.36	97.67	93.58	36.65	99.34
E.2	6.76	6.72	97.83	94.06	36.97	99.36
E.3	5.97	5.93	97.54	93.18	36.60	99.39
L1.1	6.81	6.68	97.50	93.30	41.23	98.03
L1.2	6.01	5.92	97.53	93.32	41.87	98.45
L1.3	7.92	7.74	97.51	93.47	40.84	97.72
L2.1	5.95	5.88	97.64	93.61	41.75	98.94
L2.2	6.51	6.41	97.62	93.63	41.49	98.54
L2.3	6.64	6.54	97.64	93.57	40.35	98.47
L3.1	6.82	6.77	97.87	94.04	37.51	99.23
L3.2	6.84	6.77	97.25	92.21	37.59	98.96
L3.3	6.10	6.03	97.78	93.90	37.51	98.94
L4.1	6.87	6.81	97.81	93.99	39.18	99.09
L4.2	6.65	6.59	97.73	93.77	38.53	99.10
L4.3	6.49	6.44	97.70	93.68	38.44	99.22
L5.1	6.60	6.56	97.78	93.87	39.14	99.30
L5.2	6.86	6.81	97.63	93.41	38.45	99.24
L5.3	5.95	5.88	97.82	93.99	38.70	98.94
L6.1	6.59	6.53	97.72	93.61	41.93	99.20
L6.2	6.56	6.49	97.75	93.82	39.46	98.95
L6.3	6.53	6.47	97.86	94.08	41.01	99.08
P.1	6.77	6.69	97.64	93.67	41.37	98.90
P.2	6.02	5.94	97.59	93.46	40.62	98.64
P.3	6.84	6.76	97.71	93.78	39.68	98.72
A.1	6.30	6.24	97.53	93.30	40.87	98.93
A.2	6.34	6.26	96.64	90.72	40.93	98.76
A.3	6.43	6.36	97.72	93.83	40.22	98.92

Raw Base (G): Total base count of raw sequencing data, calculated by multiplying the number of sequencing reads by the sequencing length, expressed in gigabases (G). Clean Base (G): Volume of high-quality data after filtering, calculated by multiplying the number of filtered sequencing reads by the read length, expressed in gigabases (G).

Clean Q20 (%): Proportion of bases with a Phred quality score greater than 20 in the Clean Base data. Clean Q30 (%): Proportion of bases with a Phred quality score greater than 30 in the Clean Base data. Clean GC (%): Proportion of guanine (G) and cytosine (C) bases in the total Clean Base data. Effective (%): Percentage of Clean Base relative to Raw Base.

TABLE S2 Metagenome assembly results of intestinal bacteria of *S. litura*

Sample ID	Total len(bp)	Scaftigs num	Average len(bp)	N50 len(bp)	N90 len(bp)	Max len(bp)
E.1	402,649,500	73,093	5,508.73	10,797	2,514	123,288
E.2	402,360,142	76,515	5,258.58	10,082	2,376	131,849
E.3	401,557,999	79,178	5,071.59	9,488	2,284	131,857
L1.1	386,938,239	163,834	2,361.77	3,398	1,065	133,970
L1.2	376,365,908	198,187	1,899.04	2,540	884	66,595
L1.3	396,857,353	152,504	2,602.27	3,851	1,173	93,857
L2.1	377,211,924	201,584	1,871.24	2,497	859	133,679
L2.2	386,755,948	182,098	2,123.89	2,958	972	53,511
L2.3	399,473,842	155,552	2,568.10	3,815	1,147	66,628
L3.1	411,594,906	78,460	5,245.92	10,149	2,366	514,399
L3.2	411,228,796	78,566	5,234.18	10,123	2,347	377,554
L3.3	411,186,479	83,147	4,945.30	9,392	2,221	390,114
L4.1	410,693,229	86,405	4,753.12	8,970	2,088	514,527
L4.2	406,964,466	85,308	4,770.53	9,225	2,073	514,526
L4.3	409,856,747	81,396	5,035.34	9,886	2,236	514,526
L5.1	406,074,757	82,470	4,923.91	9,076	2,236	514,526
L5.2	411,723,190	80,630	5,106.33	10,100	2,258	514,406
L5.3	409,717,735	88,392	4,635.24	8,480	2,059	514,526
L6.1	402,180,341	107,631	3,736.66	6,583	1,592	514,526
L6.2	408,108,012	90,734	4,497.85	8,669	1,919	514,526
L6.3	402,849,336	104,389	3,859.12	6,995	1,633	514,406
P.1	369,401,399	177,825	2,077.33	2,852	963	34,537
P.2	375,381,881	164,847	2,277.15	3,251	1,035	48,938
P.3	390,183,919	131,124	2,975.69	4,610	1,330	63,778
A.1	368,730,767	180,074	2,047.66	2,827	942	48,572
A.2	383,850,425	162,986	2,355.11	3,387	1,063	397,842
A.3	381,486,459	154,802	2,464.35	3,618	1,107	42,547

Total len (bp): Total length of assembled scaftigs. Scaftigs num: Total number of assembled scaftigs. Average len (bp): Mean length of scaftigs. N50 len (bp): Length of the shortest scaftig in the sorted-by-descending-length list at which the cumulative length covers 50% of the total assembly length. N90 len (bp): Length of the shortest scaftig in the sorted-by-descending-length list at which the cumulative length covers 90% of the total assembly length. Max len (bp): Length of the longest assembled

19 scaftig.

Table S3 Statistics of basic information of gene catalog

ORFs NO.	Total_Len.(Mbp)	Average_Len.(bp)	GC_Percent
266,054	88.31	331.91	45.27

ORFs_NO.: Number of genes in the gene catalogue. Total_Len. (Mbp): Total length of genes in the gene catalogue, expressed in megabase pairs (Mbp). Average_Len. (bp): Average length of genes in the gene catalogue, expressed in base pairs (bp). GC_Percent: Overall GC content of the predicted genes in the gene catalogue.

Table S4 Microbial sources of key detoxification and metabolic enzymes.

Gene ID	Enzyme	Genus name
A.1_13000, A.1_17596, A.1_24398, A.1_32471, A.1_3991, A.1_45880, A.1_59452, A.1_72535, A.1_81105, A.2_25692, A.2_58724, A.2_80194, L2.3_52804	XDH	Gammaproteobacteria
A.2_48325, A.3_48323, A.3_64114	NDK	Enterobacter
L1.1_84163	UCK	Enterobacter
L1.1_12743, L1.1_71925, L1.1_93163	ADH5	Enterococcus
L1.1_12743, L1.1_71925, L1.1_93163	frmA	Enterococcus
L1.2_49071 L1.3_16054 L1.3_28232 L1.3_74079 L1.3_89868 L2.1_67158 L2.1_76537 L2.2_102180	GST	Pseudomonas
A.2_30751, A.2_51214, A.2_64149, A.2_70191 L2.1_109868		Enterobacter
A.1_63684 L1.3_24266		Escherichia
E.1_27281, A.1_65474, L1.3_41271	UGT	Escherichia

Supplementary Figures

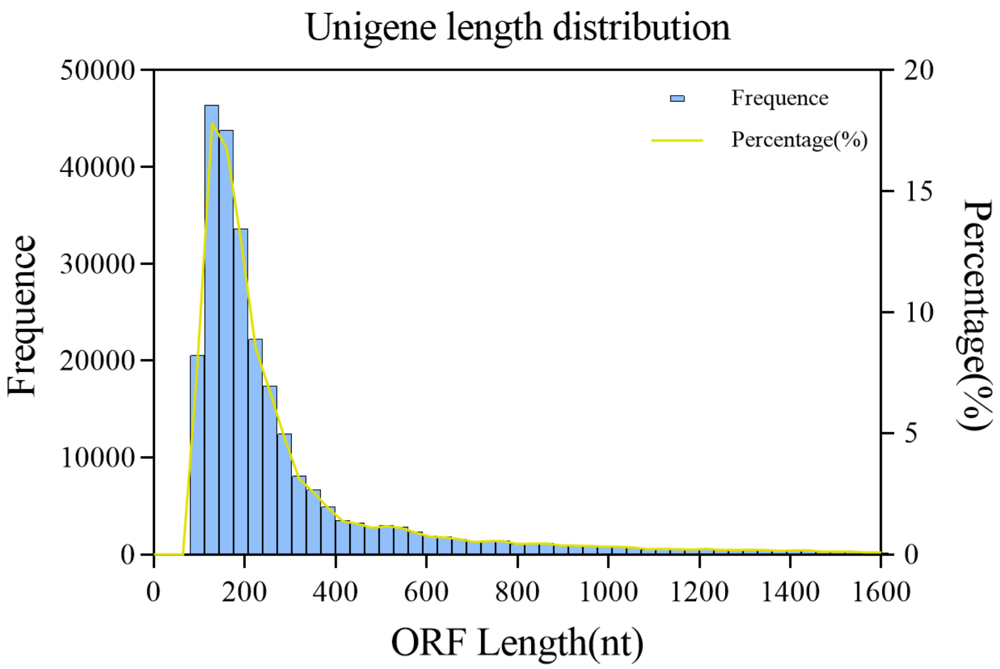

FIG S1 Unigene length distribution statistics.
